# Influence of wide band gap oxide substrates on the photoelectrochemical properties and structural disorder of CdS nanoparticles grown by the successive ionic layer adsorption and reaction (SILAR) method

**DOI:** 10.3762/bjnano.6.231

**Published:** 2015-11-30

**Authors:** Mikalai V Malashchonak, Alexander V Mazanik, Olga V Korolik, Еugene А Streltsov, Anatoly I Kulak

**Affiliations:** 1 Faculty of Chemistry, Belarusian State University, Nezalezhnastsi Av. 4, 220030 Minsk, Belarus; 2 Faculty of Physics, Belarusian State University, Nezalezhnastsi Av. 4, 220030 Minsk, Belarus; 3 Institute of General and Inorganic Chemistry, National Academy of Sciences of Belarus, Surganov St. 9/1, 220072 Minsk, Belarus

**Keywords:** CdS, nanoparticles, Raman spectroscopy, semiconductor photoelectrochemistry, wide band gap oxide

## Abstract

The photoelectrochemical properties of nanoheterostructures based on the wide band gap oxide substrates (ZnO, TiO_2_, In_2_O_3_) and CdS nanoparticles deposited by the successive ionic layer adsorption and reaction (SILAR) method have been studied as a function of the CdS deposition cycle number (*N*). The incident photon-to-current conversion efficiency (IPCE) passes through a maximum with the increase of *N*, which is ascribed to the competition between the increase in optical absorption and photocarrier recombination. The maximal IPCE values for the In_2_O_3_/CdS and ZnO/CdS heterostructures are attained at *N* ≈ 20, whereas for TiO_2_/CdS, the appropriate *N* value is an order of magnitude higher. The photocurrent and Raman spectroscopy studies of CdS nanoparticles revealed the occurrence of the quantum confinement effect, demonstrating the most rapid weakening with the increase of *N* in ZnO/CdS heterostructures. The structural disorder of CdS nanoparticles was characterized by the Urbach energy (*E*_U_), spectral width of the CdS longitudinal optical (LO) phonon band and the relative intensity of the surface optical (SO) phonon band in the Raman spectra. Maximal values of *E*_U_ (100–120 meV) correspond to СdS nanoparticles on a In_2_O_3_ surface, correlating with the fact that the CdS LO band spectral width and intensity ratio for the CdS SO and LO bands are maximal for In_2_O_3_/CdS films. A notable variation in the degree of disorder of CdS nanoparticles is observed only in the initial stages of CdS growth (several tens of deposition cycles), indicating the preservation of the nanocrystalline state of CdS over a wide range of SILAR cycles.

## Introduction

Quantum dot sensitized solar cells (QDSSCs) utilize light absorbed by semiconductor nanoparticles (CdS, CdSe, CdTe, PbS, etc.) deposited on wide band gap oxide (WBGO) scaffolds (TiO_2_, ZnO, In_2_O_3_) which act as a photoanode [1–10]. In the last decade, the SILAR method, based on specific ionic adsorption and reaction of cations and anions [11–13], has been widely used to form chalcogenide semiconductor nanoparticles (NPs) in such structures [14–15]. This technique involves successive immersion of the electrode–substrate into solutions containing cations and anions of required elements (e.g., Cd^2+^ and S^2−^), thus the number of deposition cycles determines the size of the formed NPs [4–5]. Apart from its simplicity, the SILAR method provides an intimate contact of the deposit with a substrate, facilitating an efficient transfer of photogenerated carriers into an oxide matrix. Since the SILAR method allows the formation of semiconductor nanoparticles directly on a substrate, it is reasonable to expect a significant impact of the latter on the NP properties.

Despite the widespread applications of the SILAR method to form CdS NPs on an oxide substrate, its influence on the quantum efficiency of photoelectrochemical processes and the structural disorder of CdS NPs with an increasing number of SILAR cycles (*N*) is still unclear. The aim of this study was to investigate the foregoing features to make the SILAR technique more flexible and predictable for QDSSC application.

Along with the photoelectrochemical investigation, Raman spectroscopy was used to characterize CdS NPs. Raman spectroscopy in the simplest case can be applied as a type of phase analysis [16–17]. At the same time, in the general case, Raman spectra are determined by both phonon and electron spectra of the system under study, electron–phonon coupling, material defectiveness, and elastic stresses, providing therefore valuable information about the NP properties. Nevertheless, to the best of our knowledge, there are no thorough studies with a systematic comparison of Raman spectra for the SILAR-grown NPs illustrating their dependence on the number of SILAR cycles and the wide band gap oxide substrate used.

## Results and Discussion

### SEM, BET, and XRD studies of ZnO, TiO_2_, and In_2_O_3_ electrodes

**Figure 1:**
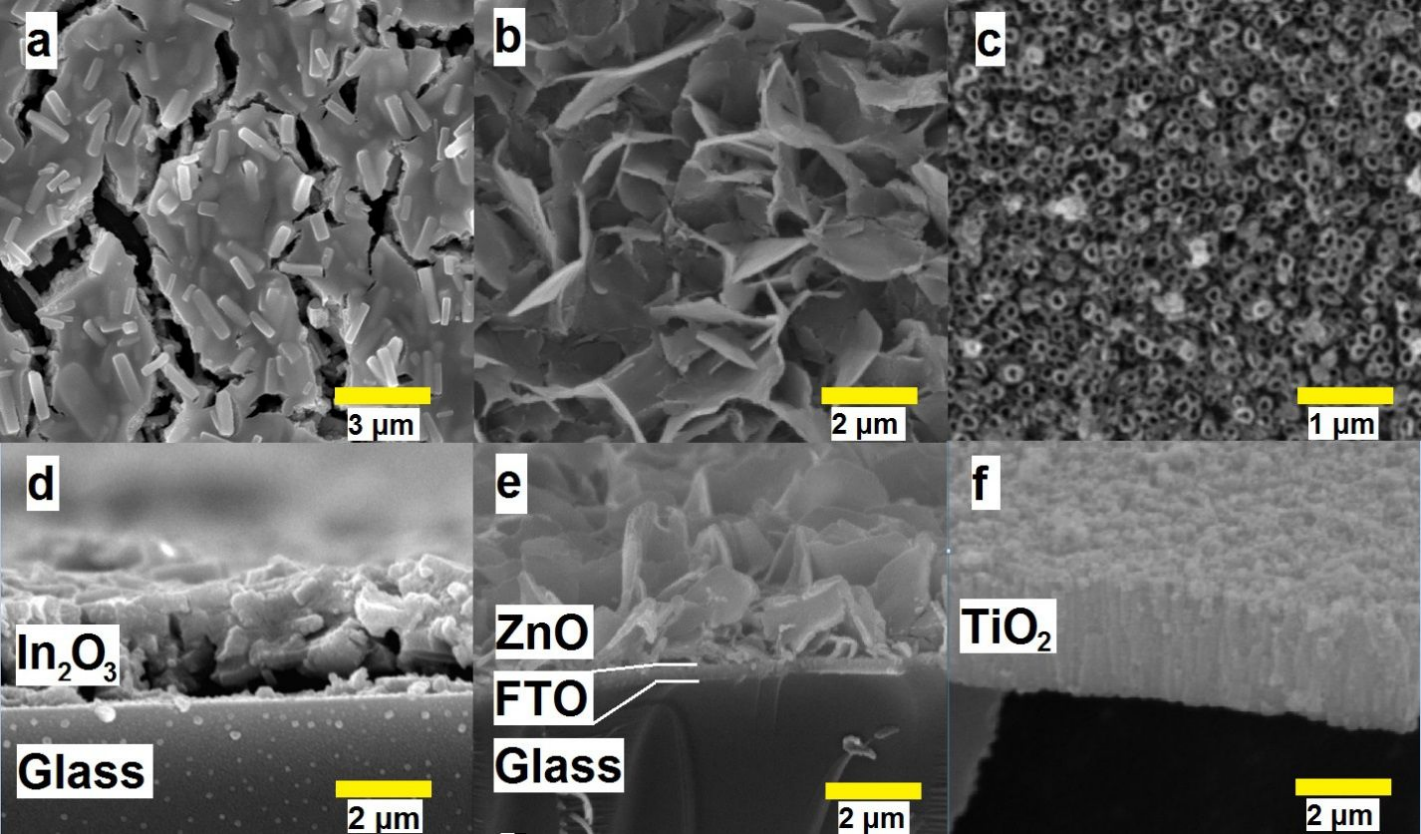
SEM images of In_2_O_3_ mesoporous films (a,d), ZnO platelet crystallites (b,e) and anodic TiO_2_ nanotubes (c,f).

Top and cross-sectional scanning electron microscopy (SEM) images of indium, zinc, and titanium oxide prepared films are shown in Figure 1. The In_2_O_3_ films (Figure 1a,d) have a uniform thickness and are characterized by a more dense packing of the grains as compared to the ZnO deposit, which consists of plate-like crystallites with approximately 100 nm thickness and a lateral size of 1–2 μm (Figure 1b,e). The length of the anodic TiO_2_ nanotubes is about 2 μm (Figure 1f), and their inner diameter is several tens of nanometers (Figure 1c). The calculated Brunauer–Emmelt–Teller (BET) specific surface area is as large as 119 ± 8 m^2^/g for In_2_O_3_ and 105 ± 12 m^2^/g for ZnO. The specific surface area estimated from SEM images for the TiO_2_ nanotube array is about 20 m^2^/g.

X-ray diffraction (XRD) analysis demonstrates that In_2_O_3_, ZnO, and TiO_2_ crystallize in the cubic, hexagonal, and anatase modifications, respectively, after the heat treatment.

### Photoelectrochemical study of ZnO/CdS, TiO_2_/CdS, and In_2_O_3_/CdS heterostructures

**Figure 2:**
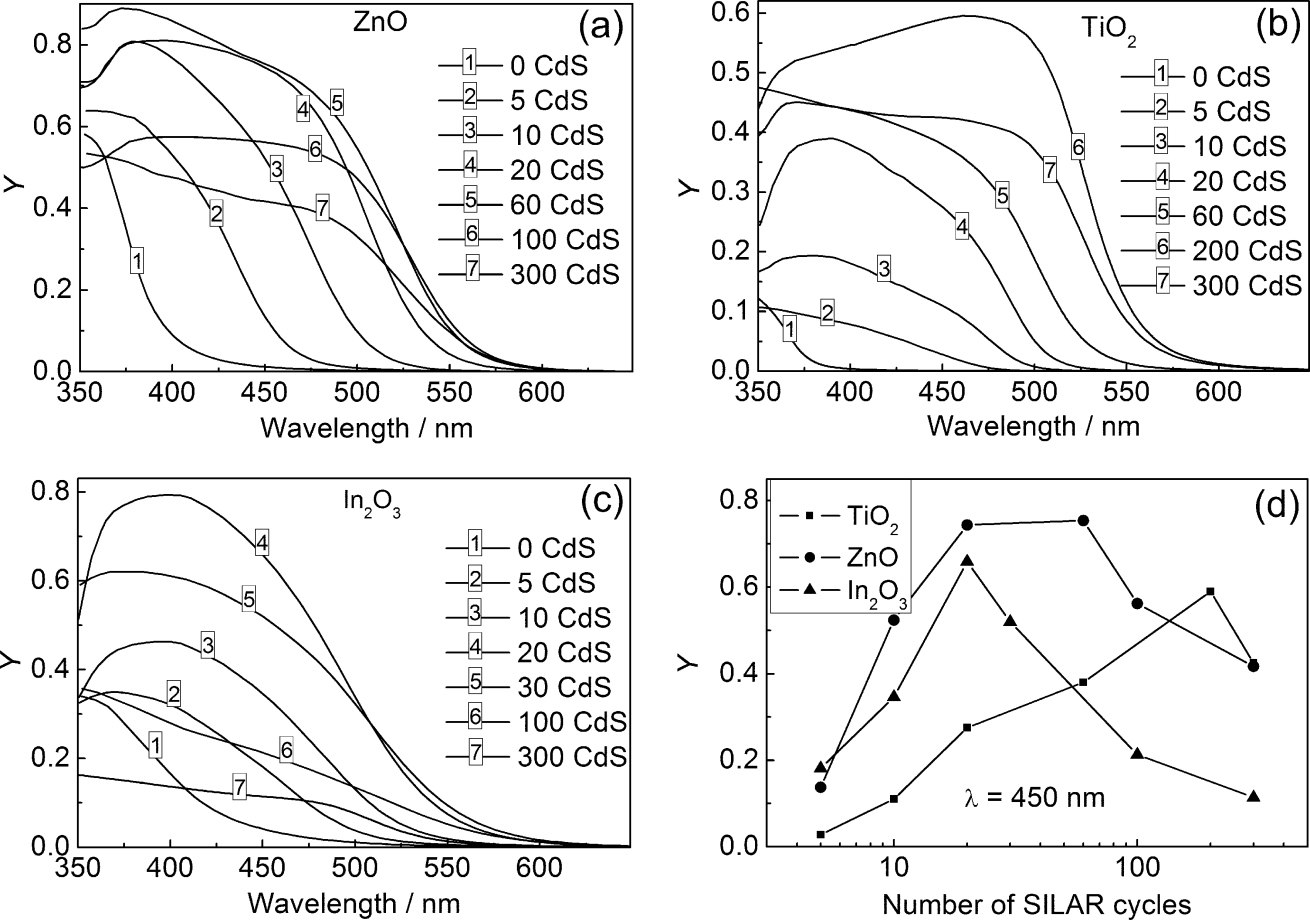
Spectral dependence of IPCE for ZnO/CdS (a), TiO_2_/CdS (b), and In_2_O_3_/CdS heterostructures (c). IPCE at λ = 450 nm as a function of the number of SILAR deposition cycles (d). Electrode potential: −0.2 V.

The incident photon-to-current conversion efficiency (IPCE) spectra of the heterostructures are presented in Figure 2. The conduction band level of CdS is located relatively higher than that of the WBGO. Therefore, the photocurrent observed for the heterostructures is attributed to the charge carrier photogeneration in CdS NPs followed by the transfer of photoelectrons into the oxide matrix, whereas the photoholes are trapped by sulphite ions, SO_3_^2−^. Sulfide ions S^2−^ in the electrolyte reduce the solubility of CdS and facilitate its reprecipitation during the photocorrosion process.

It should be noted that the oxide (ZnO, TiO_2_, In_2_O_3_)/CdS heterostructures are characterized by relatively high IPCE values of up to 60–90% at a photon energy of about 3 eV. This is due to the intimate contact of SILAR-deposited CdS nanoparticles with the substrate, ensuring improved electron transfer from the NPs to the oxide matrix.

For all types of WBGO substrates the dependence of IPCE (*Y*) on the number of SILAR cycles passes through a maximum (Figure 2d). The increase in IPCE in the range of small *N* is naturally related to the increase in optical absorption, whereas the IPCE decrease at larger *N* indicates the rising influence of nonequilibrium charge carrier recombination due to the increase of the distance which the photoelectrons must overcome to reach the substrate surface. The position of the maximum in the IPCE–*N* curves is noticeably different for the different WBGOs (Figure 2d). Particularly, for TiO_2_/CdS, the maximum is shifted towards larger *N*, and the IPCE values obtained are smaller as compared with the ZnO/CdS and In_2_O_3_/CdS heterostructures. This result can be attributed to the fact that the specific area of the TiO_2_ nanotube array is significantly smaller in comparison with the ZnO and In_2_O_3_ films. Therefore, the light absorption in sulfide is the main factor limiting the photocurrent for the TiO_2_/CdS films, thus the increase in the amount of sulfide determines the IPCE growth over a wide range of *N*. At the same time, as will be shown later, the CdS NPs grown on the TiO_2_ surface possess a minor degree of disorder as compared with the ZnO/CdS and In_2_O_3_/CdS heterostructures. Therefore, the charge carrier recombination slightly affects the photocurrent value in the TiO_2_/CdS system. On the contrary, in the case of the In_2_O_3_/CdS heterostructures, the disorder of the CdS NPs is maximal (see below), and the decrease in IPCE with increasing *N* is observed beginning with a small number (*N* ≥ 20) of SILAR cycles. From a practical point of view, it is important that the optimal value of the number of SILAR cycles is at least a few tens and significantly depends on the WBGO substrate used.

**Figure 3:**
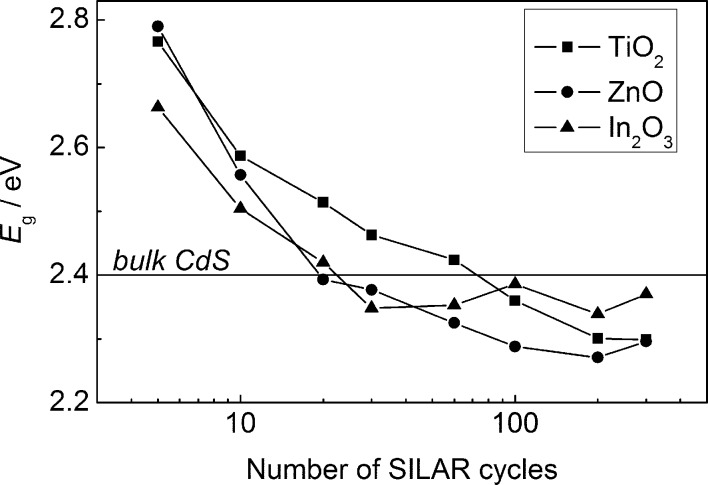
CdS band gap *E*_g_ dependence on the number of SILAR deposition cycles.

The amount of deposited CdS has a significant impact on the shape of the photocurrent spectra of the heterostructures. This is characterized by a red shift of the photocurrent edge with increasing *N* (Figure 2a–c). The Tauc plot for direct optical transitions (*Yh*ν)^2^–*h*ν demonstrates that the band gap of CdS (*E*_g_) significantly decreases as the number of SILAR deposition cycles increases. This holds within the range of *N* = 5–300: from 2.79 to 2.30 eV for ZnO/CdS, from 2.77 to 2.30 eV for TiO_2_/CdS, and from 2.66 to 2.37 eV for In_2_O_3_/CdS (Figure 3). The values of *E*_g_ evaluated from the (*Yh*ν)^2^*–h*ν dependence for CdS nanoparticles exceed the *E*_g_ value of 2.4 eV for bulk cadmium sulfide at *N* ≤ 10 for ZnO/СdS, at *N* ≤ 20 for In_2_O_3_/СdS, and at *N* ≤ 60 for TiO_2_/СdS, indicating an electron-quantum-confinement effect. At the same time, at large *N* the *E*_g_ values become noticeably smaller than 2.4 eV. The reasons for this effect observed for ZnO/CdS were analyzed by Rabinovich and Hodes [15] as well as in our previous work [18]. As it is shown in [18], the sub-band gap (SBG) photoelectrochemical processes take place in ZnO/CdS heterostructures; their IPCE can reach large values (up to 25%) for optimized CdS NP and substrate morphology. The red shift in the photocurrent spectra with the increased number of SILAR cycles as well as the high efficiency of the SBG processes are likely to be due to light scattering from inhomogeneities in ZnO/CdS at the comparable wavelength [18].

**Figure 4:**
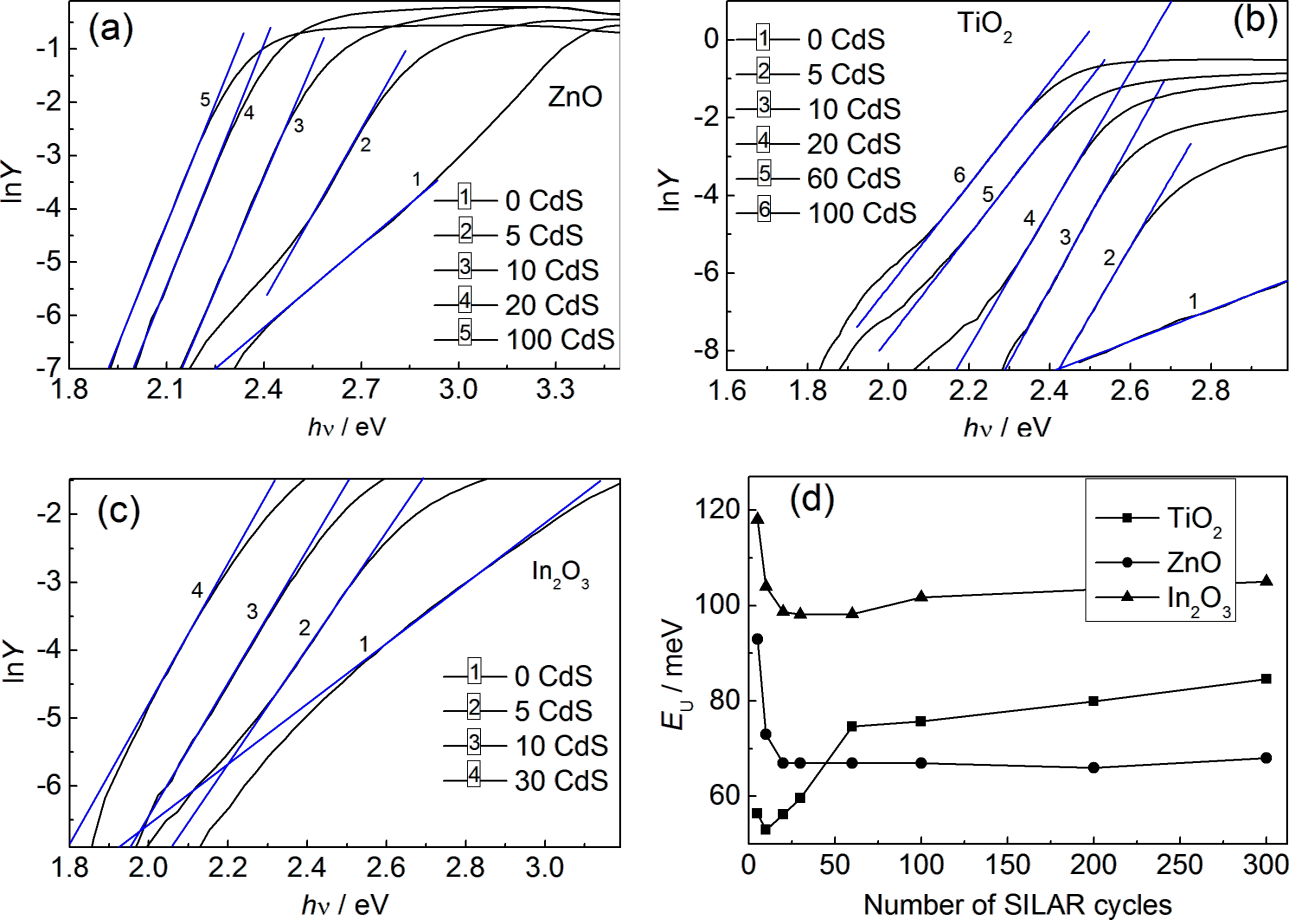
Spectral dependence of IPCE in ln*Y*–*h*ν coordinates for ZnO/CdS (a), TiO_2_/CdS (b), and In_2_O_3_/CdS heterostructures (c). Urbach energy for CdS NPs as a function of the number of SILAR cycles (d).

There is a long-wavelength region of the photocurrent spectra for heterostructures of all three types (ZnO/СdS, In_2_O_3_/СdS, and TiO_2_/СdS), where IPCE depends exponentially on the excitation energy (Figure 4a–c), i.e., the Urbach law [19] takes place.

It should be noted that the calculated Urbach energy (*E*_U_) values given below should be considered with reasonable skepticism due to the fact that *E*_U_ is derived from the photocurrent spectrum range with a small absorption coefficient where the light scattering extending an optical path length is able to significantly influence the spectral dependence of the number of absorbed quanta [18]. As our additional experiments have revealed, the shape of the photocurrent spectrum in this case depends on the film morphology, which determines the efficiency of light scattering processes. As a result, the *E*_U_ value reflects not only the spectrum of electronic states, but is determined also by the microstructure of the system under study. At the same time, the Urbach energy enables one to track the variation of structural disorder with *N*, because according to the obtained SEM data, variation of the number of SILAR cycles over a wide range has no appreciable impact on the film morphology and, hence, light scattering efficiency.

The estimated Urbach energy for CdS nanoparticles as a function of *N* is given in Figure 4d. Large *E*_U_ values (tens to hundreds of meV) indicate a high degree of structural disorder in CdS nanoparticles, which significantly depends on the oxide substrate. For *N* ranging from 60 to 300, *E*_U_ values are practically constant for ZnO/СdS but demonstrate a slight growth for In_2_O_3_/СdS and TiO_2_/СdS. This fact points out the preservation of the degree of disorder for CdS NPs over a wide range of *N*.

### Raman spectroscopy study of ZnO/CdS, TiO_2_/CdS, and In_2_O_3_/CdS heterostructures

**Figure 5:**
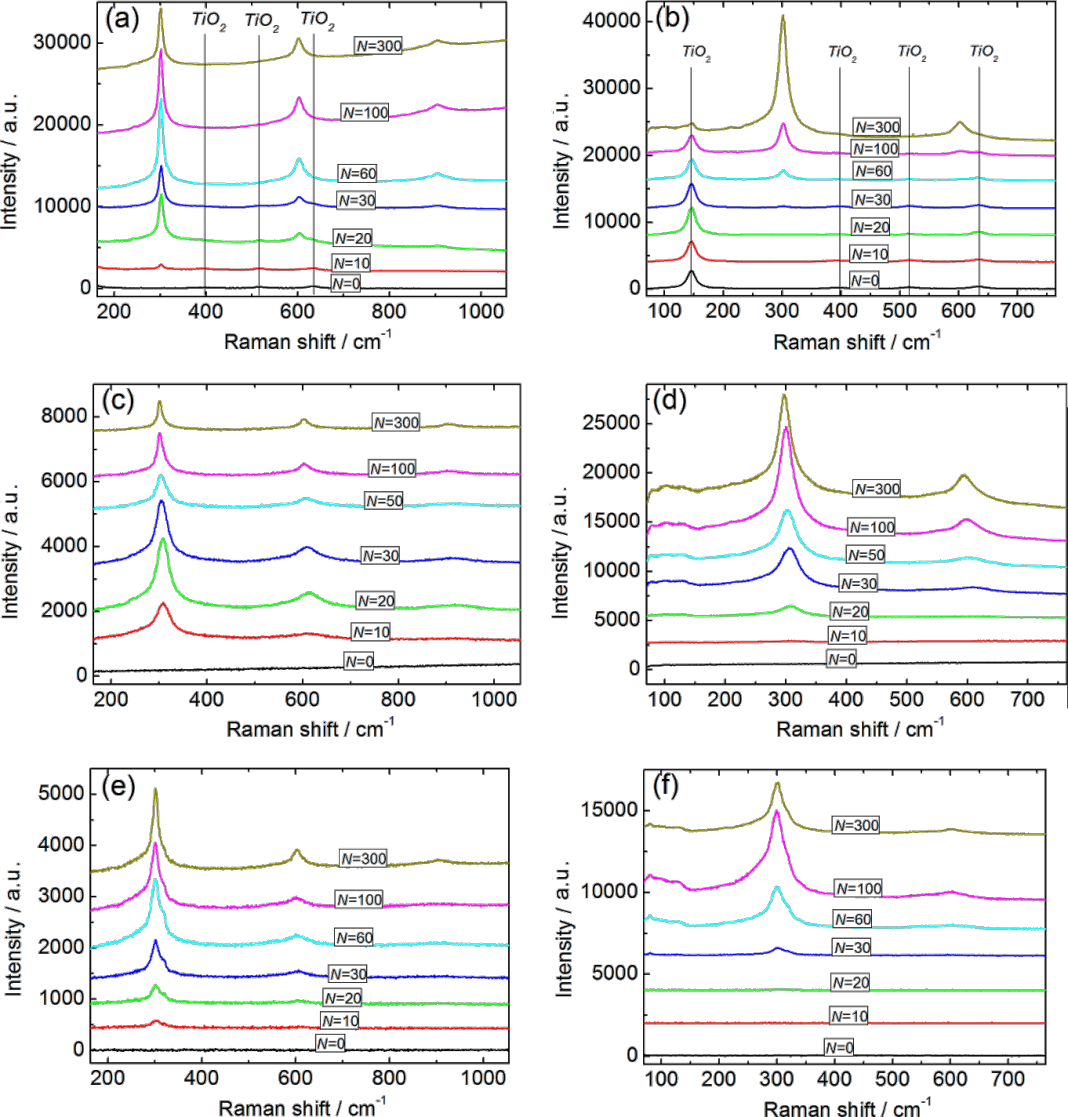
Raman spectra for TiO_2_/CdS (a,b), ZnO/CdS (c,d), and In_2_O_3_/CdS (e,f) films. Excitation: 473 nm/25 μW (a,c,e) and 532 nm/200 μW (b,d,f). The bands associated with TiO_2_ [21] are marked by vertical lines (a,b). The curves are arranged along the *y* axis for convenience.

The Raman spectra of ZnO/CdS, TiO_2_/CdS, and In_2_O_3_/CdS films are shown in Figure 5. In the studied range of Raman shifts, the band at ≈300 cm^−1^ corresponded to scattering by CdS longitudinal optical (LO) phonons [20] and also two of its overtones (≈600 and ≈900 cm^−1^) are observed after realization of the SILAR process, independent of the WBGO nature. Since the band gap energies for In_2_O_3_, TiO_2_, and ZnO are much higher than the energy of the Raman spectra exciting quanta, the signals from the WBGOs have low intensities in comparison with the signal from cadmium sulfide and are almost invisible at the low excitation power used (except for TiO_2_, for which the Raman peak intensity is somewhat higher than that for In_2_O_3_ and ZnO).

**Figure 6:**
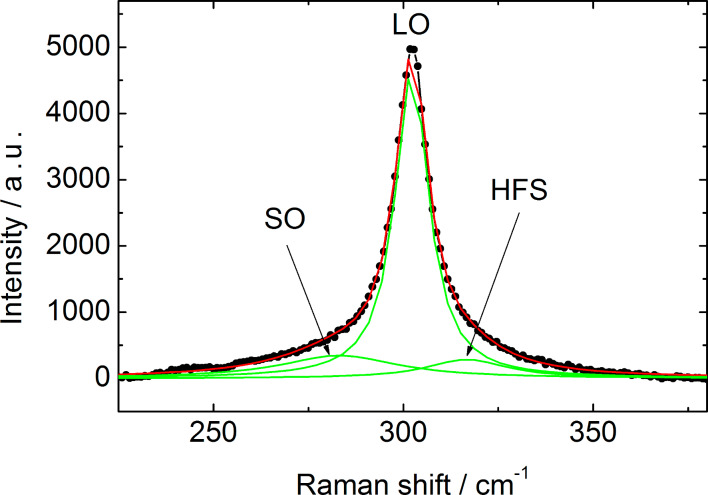
Approximation of Raman spectrum for TiO_2_/CdS film (*N* = 30) in the vicinity of the CdS LO band by superposition of the Lorentz lines.

The approximation of the Raman spectra in the vicinity of the CdS LO band by one Lorenz line results in a significant discrepancy between the experimental and fitted contours. Along with the main band one can observe additional components in the spectral ranges corresponding to both larger and smaller Raman shifts (Figure 6).

The observed low-energy shoulder in the Raman spectra of CdS NPs in the general case may be due to the following factors: phonon confinement leading to relaxation of the *k* = 0 selection rule for single-phonon scattering (*k* is a phonon wavevector) and an asymmetric low-energy LO band broadening [22]; scattering by disorder-activated zone-edge (DAZE) phonons [23]; or scattering by surface optical (SO) phonons [24]. As is known, the influence of phonon confinement on the line shape in Raman spectra increases with dispersion, ω(*k*), of optical phonons. For CdS, where the LO phonon frequency ω slightly depends on the wavevector, the impact of phonon confinement on the shape of spectral lines becomes significant only when the nanoparticles diameter is less than 2 nm [25]. However, in our experiments, even at *N* = 5 the NP diameter, estimated from the photosensitivity edge position, exceeds 3 nm [26]. The band associated with scattering by DAZE phonons is observed at ≈293 cm^−1^ for CdS nanoparticles [27], that is, at much higher than the low-energy shoulder in our experiments (265–285 cm^−1^ depending on the WBGO). Thus, the contribution of the surface optical phonons seems to be the most likely reason for the low-energy shoulder observed in the Raman spectra.

The nature of the components in the Raman spectra in the region of the shifts exceeding the CdS LO band position is still under discussion. As the early pioneering work revealed [20], the bands at 328 cm^−1^ and 347 cm^−1^ correspond to two-phonon scattering in CdS. Nevertheless, it behooves us to consider that in our case these components are not only associated with the two-phonon processes, in particular because the relative probability of two-phonon scattering in nanoparticles is smaller than in the bulk material. In a prior work [28], the signal in this spectral region (according to this work it will be denoted as a high-frequency shoulder (HFS)) was tentatively related to the undercoordinated atoms on the surface of the nanoparticle.

**Figure 7:**
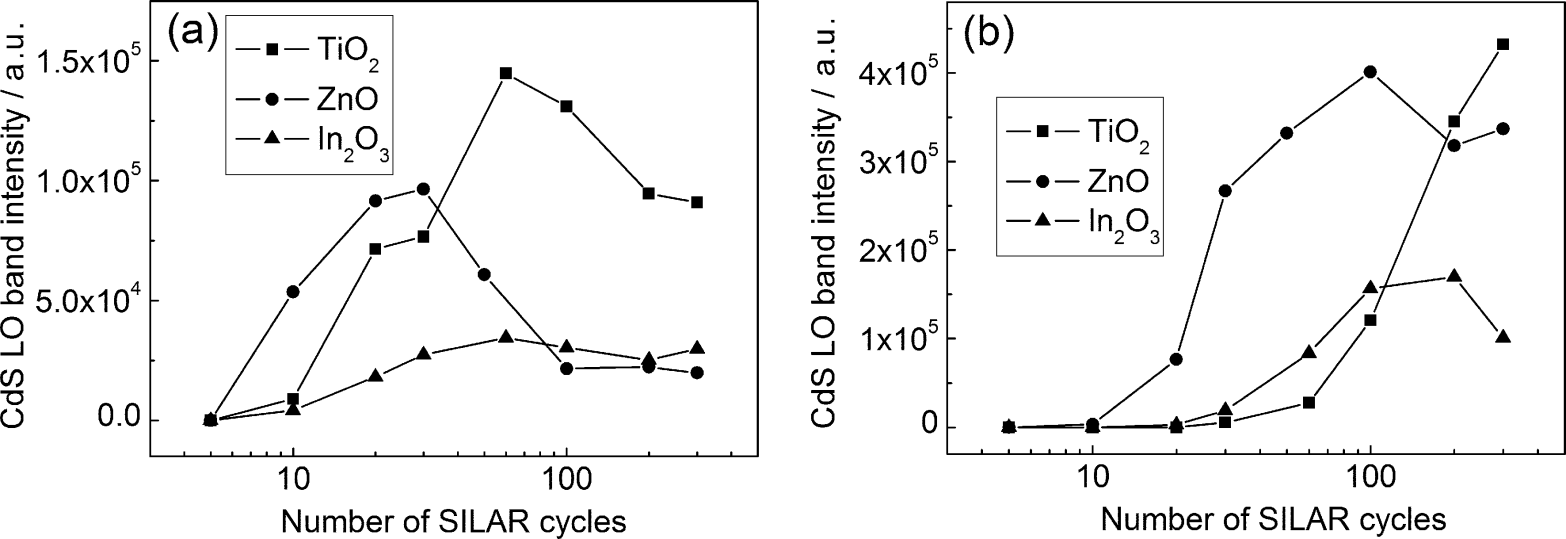
Intensity of CdS LO band as a function of the SILAR cycle number. Excitation: 473 nm/25 μW (a) and 532 nm/200 μW (b).

The dependence of the CdS LO band intensity on the number of SILAR cycles demonstrates a sharp increase in the signal at small *N* followed by a slight decrease (Figure 7). In the case of 532 nm excitation, a sharp increase in the signal intensity is observed at larger *N* values. This result is in agreement with the above-mentioned *E*_g_(*N*) dependence. Indeed, the intensity of the Raman signal reaches its maximum when the energy of exciting photons equals the band gap of the semiconductor [29]. For small *N* values, due to the quantum-confinement effect, the band gap energy of CdS NPs exceeds the energy of the exciting radiation quanta, *h*ν. Thus the increase in *N* leads not only to an increase in the amount of CdS in the films, but also to the increased probability of Raman scattering. This is because the band gap energy of the NPs is reduced to *h*ν due to weakening of the quantum-confinement effect. For this reason, at an early stage in the SILAR process, the signal intensity grows faster than the CdS amount. Due to the further weakening of the quantum-confinement effect in CdS nanoparticles, at larger *N*, the photon energy exceeds *E*_g_ and the signal intensity decreases. When a green laser is used for excitation, *E*_g_ reaches *h*ν at a larger *N* (as compared to the case of 473 nm excitation), which is in agreement with the experimental results.

A rapid increase in the CdS LO band intensity for ZnO/CdS structures starts at smaller *N* values in comparison with In_2_O_3_/CdS and TiO_2_/CdS films (Figure 7). This fact coincides with the *E*_g_(*N*) dependence (Figure 3), which demonstrate that the most rapid weakening of the electron-quantum-confinement effect with increasing *N* is observed for the ZnO/CdS structures. Thus in the range 5 ≤ *N* ≤ 20 the d*E*_g_/d*N* absolute values are equal to 26 meV/cycle for ZnO/CdS and 16–17 meV/cycle for In_2_O_3_/CdS and TiO_2_/CdS.

The observed difference in the d*E*_g_/d*N* values points to a more rapid growth of CdS NPs on the ZnO surface. As the first step of SILAR process in our experiments is an adsorption of Cd^2+^ cations on the surface of WBGO, the dependence of the CdS NP growth rate on the WBGO material may be related to the difference in the Cd^2+^ adsorption efficiency. The adsorption of Cd^2+^ on the surface of ZnO nanoparticles is extremely efficient and the maximum adsorption capacity was estimated to be 387 mg/g [30]. At the same time, the adsorption capacity of the TiO_2_ surface is considerably lower and ranges within 4–60 mg/g, depending on the nanoparticle size (decreases with increasing size) [31]. It is reasonable to assume that the high surface concentration of Cd^2+^, as well as the identity of CdS and ZnO lattices, promotes a more rapid growth of the CdS nuclei on the ZnO surface at an early stage in the SILAR process. There is no published data on the adsorption of Cd^2+^ on the surface of In_2_O_3_, but we can assume that it is significantly smaller in comparison with the surface of ZnO. It is common knowledge that the process of cation adsorption on an oxide surface is largely determined by the equilibrium of protolytic reactions (surface concentration of acid–base centers) and temperature [31–32]. In particular, the increase in OH^−^ surface concentration enhances the adsorption of Cd^2+^ due to the formation of barely soluble cadmium hydroxide. ZnO and In_2_O_3_ can give a sub-monolayer of Zn(OH)_2_ and In(OH)_3_, respectively, upon contact with water. The solubility products of these compounds are 2.3·10^−17^ and 1.3·10^−37^, respectively [33]. Thus the estimated value of the OH^−^ surface concentration derived from these constants is four orders of magnitude higher in the case of Zn(OH)_2_ as compared with In(OH)_3_.

**Figure 8:**
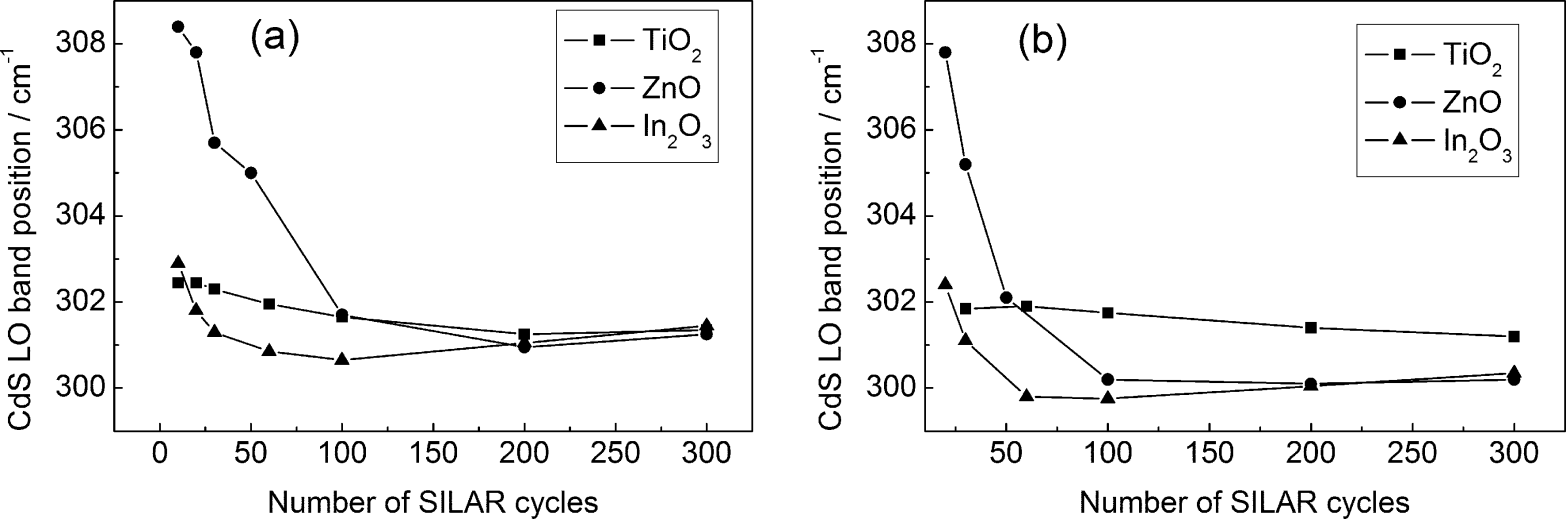
Position of CdS LO band as a function of the SILAR cycle number. Excitation: 473 nm/25 μW (a) and 532 nm/200 μW (b).

The In_2_O_3_/CdS and TiO_2_/CdS films were characterized by very close CdS LO band positions that are practically independent of both the deposition cycle number and the energy of exciting quanta (Figure 8). At the same time, for ZnO/CdS films, the LO band position is shifted by several reciprocal centimeters to the region of larger Raman shifts; at small *N* it exceeds the LO band position of bulk CdS (302–305 cm^−1^, as reported by numerous authors) and monotonically shifts towards lower values with increasing *N*. We attribute this result to the compressive lattice stresses in CdS nanoparticles (due to a mismatch of the CdS and ZnO lattice parameters) and to the relaxation of these stresses with increasing nanoparticle size in ZnO/CdS films [18].

**Figure 9:**
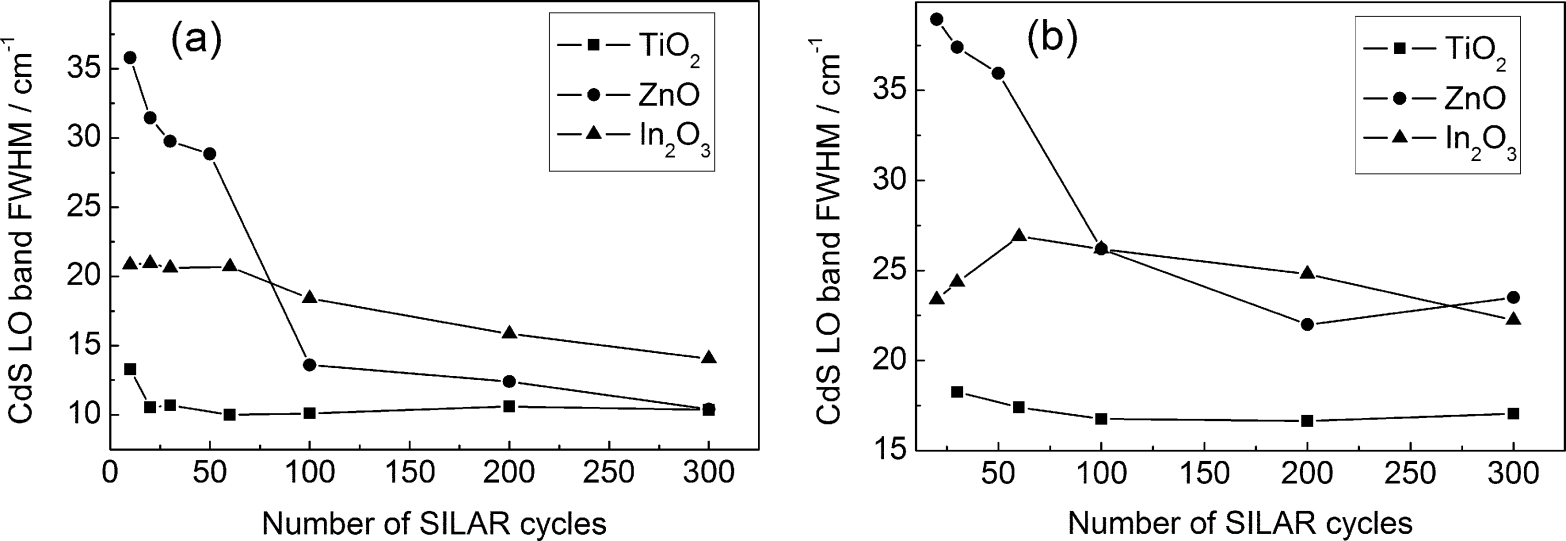
FWHM of CdS LO band as a function of the SILAR cycle number. Excitation: 473 nm/25 μW (a) and 532 nm/200 μW (b).

Figure 9 presents the CdS LO band full width at half maximum (FWHM) as a function of the deposition cycle number. It is worth noting that ZnO/CdS films are characterized by a considerably broader (by 10–20 cm^−1^) spectral width of the LO band for *N* < 100. This fact is in agreement with the presence of elastic deformations in the cadmium sulfide nanoparticles synthesized on the surface of ZnO. Indeed, the spectral width of the Raman line increases with the decreasing phonon mean-free path determined by the scattering processes from defects as well as by the decay (conversion to another two phonons) [34]. A contribution of the latter to the Raman line broadening increases with the anharmonicity of atomic oscillations. The anharmonicity contribution is more substantial in the deformed lattice. This results in the experimentally observed large spectral width of the CdS LO band for CdS/ZnO films at *N* < 100 and its decrease with a growth of *N* due to the stress relaxation.

The CdS LO band width for In_2_O_3_/CdS films is larger than for TiO_2_/CdS, which corresponds with the results of the photoelectrochemical measurements demonstrating a larger Urbach energy for the nanoparticles synthesized on the surface of indium oxide.

**Figure 10:**
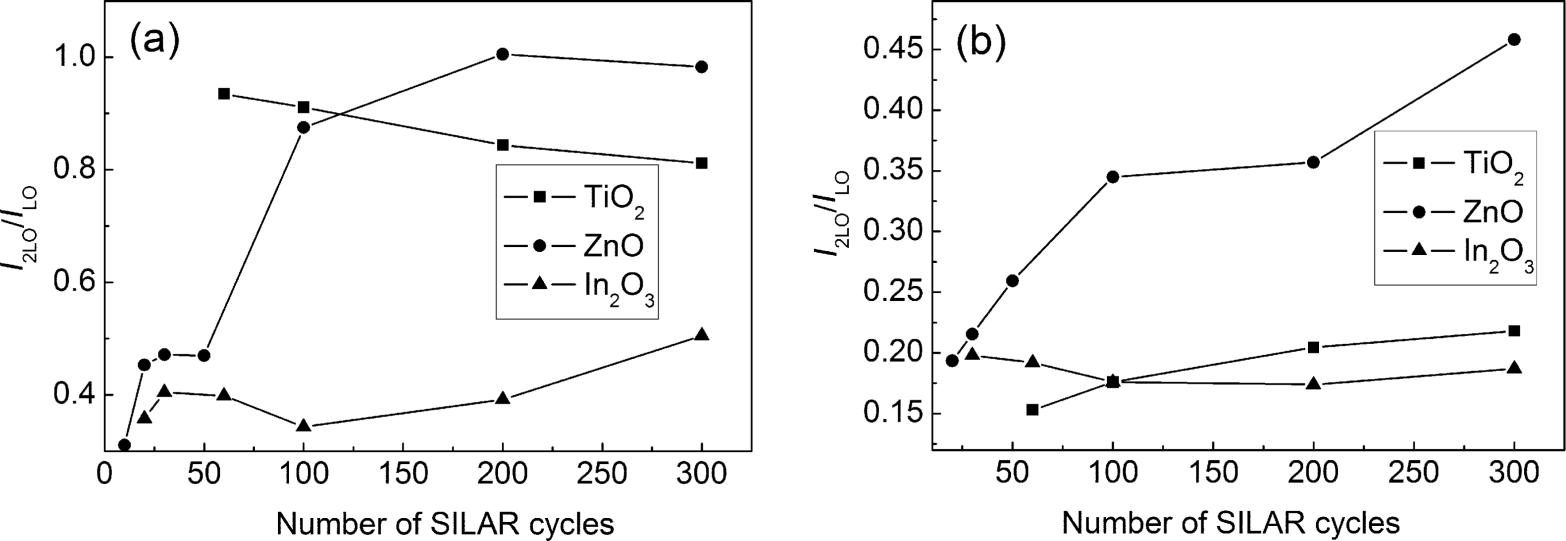
Intensity ratio of CdS 2LO and LO bands as a function of the SILAR cycle number. Excitation: 473 nm/25 μW (a) and 532 nm/200 μW (b).

The spectral widths of the CdS LO band for all films studied are 5–10 cm^−1^ wider when excited by a green laser. This fact can be explained by the Urbach energy dispersion in the ensemble of CdS nanoparticles [35]. Under excitation with a blue laser, the condition *h*ν_bl_ > *E_g_* holds, which results in resonant scattering for the entire ensemble of nanoparticles. At the same time, when using a green laser, the condition *h*ν_gr_ < *E_g_* is realized, thus resonant scattering is possible only for the most disordered CdS particles characterized by the highest Urbach energy. The dispersion of the Urbach energies of CdS nanoparticles is in agreement with the decrease in the relative intensity of the CdS *I*_2LO_ band (scattering by two LO phonons) when changing from blue to green excitation light (Figure 10).

**Figure 11:**
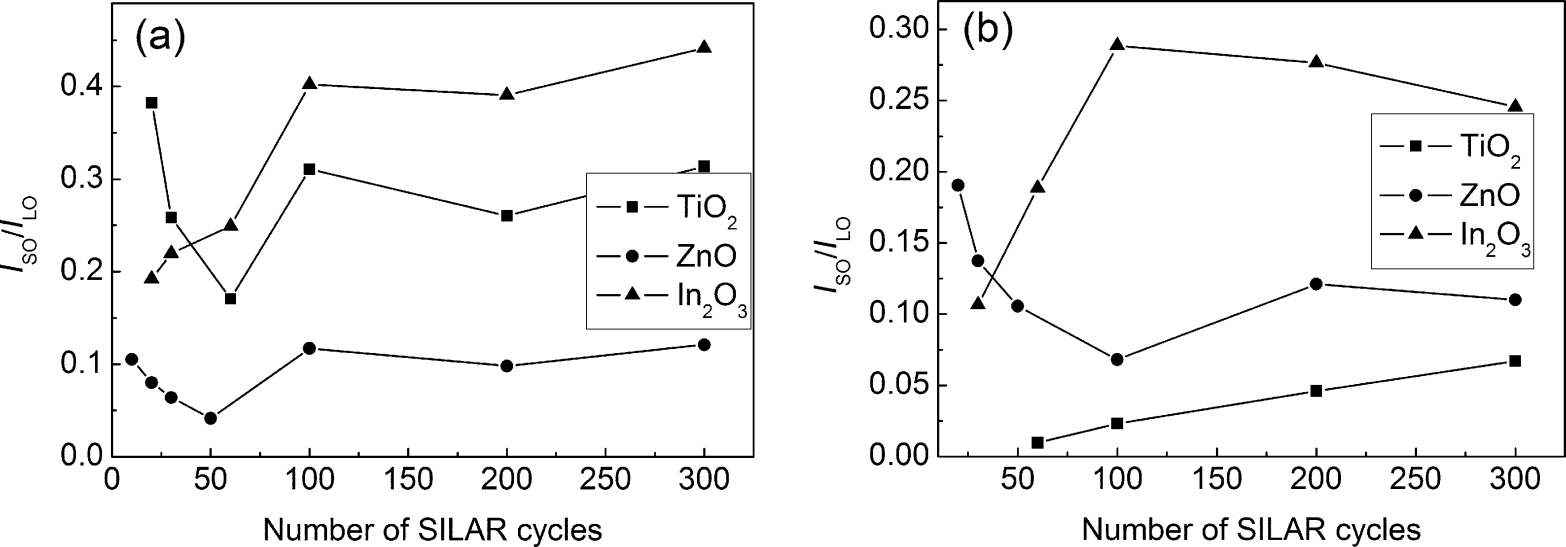
Intensity ratio of CdS SO and LO bands as a function of the SILAR cycle number. Excitation: 473 nm/25 μW (a) and 532 nm/200 μW (b).

The intensity ratio of the bands associated with CdS SO and CdS LO phonons is given in Figure 11. The maximal *I*_SO_/*I*_LO_ ratio is peculiar for the In_2_O_3_/CdS films and correlates with the maximal values of the CdS LO band spectral width and the Urbach energy for the CdS NPs formed on In_2_O_3_. A weak dependence of the *I*_SO_/*I*_LO_ ratio on the CdS deposition cycle number might seem unexpected. Meanwhile, this result correlates well with a weak dependence of the CdS LO band width and *E*_U_ on the deposition cycle number. This was also confirmed by X-ray data which show that even at *N* = 300, the size of the coherent scattering regions in CdS NPs estimated by Scherrer’s equation is below 10 nm.

## Conclusion

Similarities and distinctions in the photoelectrochemical behavior of the systems based on WBGOs (ZnO, TiO_2_, In_2_O_3_) sensitized by the SILAR-grown CdS nanoparticles have been established. Regardless of the WBGO material, at a small number of SILAR deposition cycles, the electron-quantum-confinement effect determines the spectral range of the photoelectrochemical activity. The most rapid weakening of the quantum-confinement effect with increasing *N* is observed in the ZnO/CdS structures, probably due to enhanced Cd^2+^ ion adsorption on the ZnO surface. CdS NPs grown on the WBGO surface possess a high degree of structural disorder, which was characterized by the Urbach energy, spectral width of the CdS LO band and relative intensity of CdS SO and LO bands from the Raman spectra. A higher degree of the structural disorder is peculiar for the СdS NPs crystallized on the In_2_O_3_ surface. Significant changes in the Urbach energy are observed only during the early stage of CdS deposition (*N* < 20–60). The preservation of the degree of disorder of the CdS NPs at a large number of SILAR cycles indicates that their nanocrystalline state is maintained. For all types of systems studied, the dependence of IPCE on the number of SILAR cycles, *N*, is non-monotonic and influenced by the competition of two processes: the increase in the optical absorption with *N* (i.e., with the amount of CdS) and the simultaneous enhancement of the recombination role due to the increased distance the photogenerated charge carriers must overcome. The number of *N* corresponding to the maximal IPCE values should be at least several tens and depends essentially on the substrate used due to the above-mentioned revealed distinctions in properties of nano-heterostructures based on the different WBGOs.

## Experimental

ZnO, In_2_O_3_ and TiO_2_ films were synthesized on FTO, ITO and titanium, respectively.

Mesoporous In_2_O_3_ films were prepared by spin coating of an indium hydroxide colloidal solution with subsequent heat treatment [36]. A stable indium hydroxide sol was prepared by hydrolysis of a 0.25 mol/L In(NO_3_)_3_ solution with aqueous ammonia (12%) under vigorous stirring at 0 °C to attain рН 8. The indium hydroxide precipitate was carefully washed with distilled water and sonicated after adding of 1.5 mL of concentrated nitric acid (65%) as a stabilizer per 50 mL of sol. The obtained In(OH)_3_ sol with a concentration of 200 g/L was mixed with a Pluronic F127 block copolymer (with average molecular mass of 12500 g/mol) in a ratio of 5 g of polymer per 50 mL of sol. ITO substrates were degreased and thoroughly washed in the boiling mixture of H_2_O_2_ and NH_3_ followed by spin coating with the obtained In(OH)_3_ sol at 3000 rpm for 30 s and heat treatment for 1 h at 400 °C in air.

The synthesis of the titanium dioxide nanotube arrays was carried out in a two-electrode electrochemical cell by anodization of metallic titanium with a graphite counter electrode in an aqueous electrolyte containing 1 mol/L (NH_4_)_2_SO_4_, 0.1 mol/L NH_4_F and 0.2 mol/L H_2_C_2_O_4_ with pH 2.8 (corrected with NaOH) under the electrode potential bias of 25 V for 20 h at room temperature [37]. The rate of potential bias sweep from 0 to 25 V at the initial stage was 250 mV/s. After anodization, the electrodes were immediately immersed into a 1.2 mol/L (NH_4_)_2_SO_4_ solution for 24 h then rinsed with distilled water, air-dried and heat-treated at 450 °C for 1 h in air.

Mesoporous zinc oxide films were prepared by the electrochemical cathodic deposition from water–ethanol (1:1 by volume) electrolyte containing 0.1 mol/L Zn(NO_3_)_2_, 0.1 mol/L KCl and 4 g/L poly(vinylpyrrolidone) at 50 °C [18,38]. Deposition was carried out by potentiostatic cathodic polarization of FTO electrodes at −1000 mV vs Ag/AgCl/KCl (sat.) reference electrode (+0.201 V vs SHE) for 25 min. Because of the formation of hydroxyl ions, a local increase of рН occurs, and the hydrolysis of Zn^2+^ ions is promoted and precipitation of Zn_5_(OH)_8_Cl_2_ on the electrode surface occurs. This is confirmed by the X-ray diffraction analysis [18,38]. The heating of the obtained films in air at 360 °C results in decomposition of zinc hydroxychloride with the formation of zinc oxide.

The chemical deposition of CdS on the surface of oxide (ZnO, TiO_2_, In_2_O_3_) films was carried out by successive adsorption of cadmium and sulfide ions by means of alternating immersion of the substrates into 0.1 mol/L Cd(NO_3_)_2_ and 0.01 mol/L Na_2_S aqueous solutions under vigorous stirring accompanied by intermediate rinsing with distilled water after each immersion. The duration of sample exposure in each solution and rinsing was 1 min. The cycle was repeated 5–300 times, then the sample was washed with distilled water and dried in air.

SEM images were obtained with a LEO 1455 VP scanning electron microscope. The BET surface area and porosity measurements were performed using an ASAP2020MP analyzer (Micromeritics, USA). Adsorption isotherms were obtained with N_2_ at 77 K. The surface area was calculated from the linear part of the BET plot. X-ray diffraction analysis was carried out on a Bruker D8 Advance diffractometer using Cu Kα radiation in the Bragg–Brentano geometry.

The photoelectrochemical measurements were performed according to the procedure described in [39]. A standard two-compartment three-electrode cell containing a platinum counter electrode and an Ag/AgCl/KCl (sat.) electrode as the reference electrode and controlled by a conventional programmable potentiostat was used. The photocurrent spectra were obtained using a setup equipped with a high-intensity grating monochromator (spectral resolution 1 nm), a 250 W halogen lamp, and an optical chopper. The spectral dependence of the incident photon-to-current conversion efficiency was calculated from the photocurrent spectra with a correction for the light intensity distribution at the monochromator output. The photoelectrochemical measurements were carried out in 1 mol/L Na_2_SO_3_ + 0.1 mol/L NaOH + 0.01 mol/L Na_2_S aqueous solution.

The Raman spectra were recorded at room temperature using a Nanofinder HE (LOTIS TII, Belarus–Japan) confocal spectrometer. Two solid-state lasers emitting at 473 nm (*h*ν_bl_ = 2.62 eV) and 532 nm (*h*ν_gr_ = 2.33 eV) were used for excitation of the samples. The backscattered light was dispersed on a 1800 lines/mm diffraction grating with a spectral resolution better than 1 cm^−1^ and detected using a thermostated CCD matrix with a signal acquisition time typically equal to 120 s. Calibration was performed by means of a built-in gas discharge lamp to an accuracy of ≈1 cm^−1^.
